# Heterogeneous CRISPR/Cas9 Editing of *HMOX1* Is Associated with Altered Heme–Biliverdin Metabolism and Basal Stress-Associated Transcriptional Programs in Chicken LMH Cells

**DOI:** 10.3390/ijms27167120

**Published:** 2026-08-08

**Authors:** Haonan Tang, Huaiyu Li, Yurong Tai, Xue Yang, Letian Zhang, Yuhao Ma, Ganxian Cai, Hongyang Zhao, Tong Zeng, Xiaohua Ai, Shuang He, Jiankui Wang, Zhiliang Gu, Xuemei Deng

**Affiliations:** 1 Key Laboratory of Animal Genetics, Breeding and Reproduction, Ministry of Agriculture, Beijing Key Laboratory for Animal Genetic Improvement, State Key Laboratory of Animal Biotech Breeding, China Agricultural University, Beijing 100193, China; 15178156835@163.com (H.T.); lihuaiyu96@foxmail.com (H.L.); tyd1992@126.com (Y.T.); snowyang2017@163.com (X.Y.); zzhangletian@163.com (L.Z.); jzmyh2009@163.com (Y.M.); caiganxianjcau@163.com (G.C.); zhyzyh1110@163.com (H.Z.); woshizttttt@163.com (T.Z.); aliceaxh@163.com (X.A.); hshuangcau@126.com (S.H.); jiankui@cau.edu.cn (J.W.); 2 Sanya Institute, China Agricultural University, Sanya 572025, China; 3 College of Biology and Food Engineering, Suzhou University of Technology, Suzhou 215500, China; zhilianggu88@hotmail.com

**Keywords:** *HMOX1*, heme oxygenase-1, CRISPR/Cas9, chicken LMH cells, heme metabolism, biliverdin, oxidative stress, immune signaling

## Abstract

Heme oxygenase-1 (HO-1), encoded by *HMOX1*, catalyzes the rate-limiting step of heme degradation and generates biliverdin, carbon monoxide, and ferrous iron, thereby linking heme turnover with redox regulation and stress-associated signaling. In birds, biliverdin is retained as a major heme-derived product, but the cellular consequences of *HMOX1* perturbation remain insufficiently defined. Here, CRISPR/Cas9-mediated editing was used to generate a heterogeneous *HMOX1*-edited population in Chicken hepatocellular carcinoma-derived cells. The selected sgRNA reduced HO-1 protein abundance by approximately 47%, and no detectable cleavage was observed at the seven predicted high-risk off-target loci examined. Compared with vector-control cells, *HMOX1*-edited cells exhibited intracellular heme accumulation, reduced biliverdin levels, increased oxidation-sensitive fluorescence, and reduced CCK-8 absorbance values, indicating disruption of heme–biliverdin metabolic and redox homeostasis. RNA sequencing identified 2650 differentially expressed genes, including 951 upregulated and 1699 downregulated genes. Downregulated genes were mainly enriched in immune, cytokine, MAPK/stress, and extracellular signaling-associated pathways, whereas DNA replication and cell-cycle-related genes were increased. Enrichment-term association and STRING functional-association analyses further identified a coordinated module involving *IL1B*, *JUN*, *NFKBIA*, *IRF1*, *TGFB1*, *IL10*, *CCL5*, and *PTGS2*. Independent RT-qPCR analysis confirmed selected expression trends. These findings show that heterogeneous *HMOX1* editing and reduced HO-1 abundance are associated with disruption of the avian heme–biliverdin metabolic axis and coordinated remodeling of basal immune, stress, extracellular signaling, and cell-cycle-associated transcriptional programs in Chicken hepatocellular carcinoma-derived cells.

## 1. Introduction

Heme oxygenase-1 (HO-1), encoded by *HMOX1*, catalyzes the rate-limiting step of heme degradation, producing biliverdin, CO, and free iron [1]. Through this reaction, HO-1 links heme turnover with redox regulation and stress-responsive cellular processes [2]. In mammalian systems, *HMOX1* is a well-characterized stress-inducible gene that responds to diverse oxidative and inflammatory stimuli, including heme, hypoxia, ultraviolet radiation, heat shock, nitric oxide, hydrogen peroxide, cytokines, and heavy metals [3,4,5]. HO-1 and its catalytic products have been implicated in oxidative stress responses, iron homeostasis, inflammatory regulation, and cell survival [6,7,8]. These studies have established HO-1 as an important molecular node connecting heme metabolism with cellular stress adaptation.

Compared with mammals, birds possess distinctive features of heme catabolism. In mammals, biliverdin is rapidly reduced to bilirubin by biliverdin reductase, whereas birds exhibit minimal or absent biliverdin reductase activity in many species, allowing biliverdin to accumulate as a major bile pigment and end product of heme degradation [9,10]. This physiological feature has been most visibly studied in the context of blue–green eggshell pigmentation, where biliverdin deposition contributes to shell coloration. Previous studies have identified *SLCO1B3* as a major determinant of biliverdin transport and deposition in the shell gland, and *HMOX1* expression or heme oxygenase enzymatic activity has also been associated with blue–green eggshell formation [11,12,13]. However, these studies mainly indicate associations between *HMOX1* activity and pigment-related phenotypes and do not directly define how *HMOX1* affects intracellular heme–biliverdin balance in avian cells.

Functional studies of *HMOX1* in birds remain comparatively limited. Early work on chicken *HMOX1* focused on gene cloning, promoter structure, and stress-inducible transcriptional regulation in primary chick embryo liver cells, identifying regulatory elements involved in responses to stimuli such as sodium arsenite and cobalt chloride [14,15]. More recent poultry studies have often examined HO-1 as a component of antioxidant response pathways, particularly the Nrf2/HO-1 axis, in models of ovarian aging, oxidative injury, heavy-metal exposure, or nutritional intervention [16,17,18]. Although these studies support the involvement of HO-1-associated pathways in avian stress responses, most are based on expression changes or antioxidant indices. Thus, the cellular consequences of direct *HMOX1* perturbation in avian cells remain insufficiently defined.

This unresolved question is important because altered *HMOX1*/HO-1 function may affect not only biliverdin production but also intracellular redox status and downstream transcriptional responses. In birds, where biliverdin can accumulate as a major product of heme catabolism, *HMOX1* represents a relevant entry point for investigating the relationship between heme metabolism and stress-related cellular regulation. However, direct evidence showing whether genetic perturbation of *HMOX1* alters heme–biliverdin metabolism, reactive oxygen species (ROS) levels, cell growth/viability, and immune-related gene expression programs in avian cells remains limited.

To address this question, we used CRISPR/Cas9-mediated editing to generate a partial *HMOX1*-disruption model in chicken hepatocellular carcinoma-derived (LMH) cells. We then combined metabolic assays, CCK-8 reductive/metabolic activity assessment, DCFH-DA oxidation-sensitive fluorescence measurement, and RNA-seq to evaluate the effects of *HMOX1* editing on heme–biliverdin metabolism and stress-related transcriptional responses. This study aimed to provide cellular evidence for the role of *HMOX1* in avian heme metabolism and to establish a framework for understanding how *HMOX1* perturbation may influence redox-associated and immune-related molecular programs in chicken cells.

## 2. Results

### 2.1. Heterogeneous HMOX1 Editing Generates Multiple Frameshift Alleles and Reduces HO-1 Protein Abundance in LMH Cells

To establish an *HMOX1*-edited LMH cell model, three single-guide RNA (sgRNA) expression vectors targeting exon 3 of *HMOX1*, designated sgRNA1, sgRNA2, and sgRNA3, were constructed using the PX458 backbone and verified by Sanger sequencing. Following transfection into LMH cells and fluorescence-activated sorting of GFP-positive cells, the three sgRNAs were initially evaluated by TA cloning and Sanger sequencing. Indels were identified in 23 of 37 clones for sgRNA1, 29 of 42 clones for sgRNA2, and 3 of 43 clones for sgRNA3, corresponding to clone-level Indel frequencies of 62.2%, 69.0%, and 14.0%, respectively. Because sgRNA2 produced the highest Indel frequency in this initial screening, it was selected for the generation of *HMOX1*-edited cell populations used in subsequent experiments.

Detailed sequence analysis of the initial sgRNA2 screening population revealed heterogeneous editing around the sgRNA2 target site. Among the 42 valid TA clones analyzed, 13 retained the Wild type (WT) target sequence, whereas the remaining 29 clones comprised six distinct deletion alleles, designated DE1–DE6. These alleles contained deletions of 1, 2, 17, 32, 52, and 53 bp, respectively, and represented all edited sequences identified in the screening population (Figure S1a). All six deletions altered the *HMOX1* coding frame.

Conceptual translation of the reconstructed mutant coding sequences from the native *HMOX1* initiation codon to the first in-frame stop codon yielded inferred translation products of 149, 110, 105, 107, 132, and 93 amino acids for DE1–DE6, respectively. The inferred sequences of DE2–DE6 terminated before the position corresponding to WT Asp143. In DE1, the deletion shifted the reading frame upstream of the codon encoding WT Asp143, after which translation continued through a non-native frameshift-derived sequence to a premature stop codon at amino acid 149. Consequently, none of the six inferred translation products retained the WT-encoded catalytic Asp143 or the native C-terminal region spanning amino acids 231–264 (Figure S1b).

**Figure 1:**
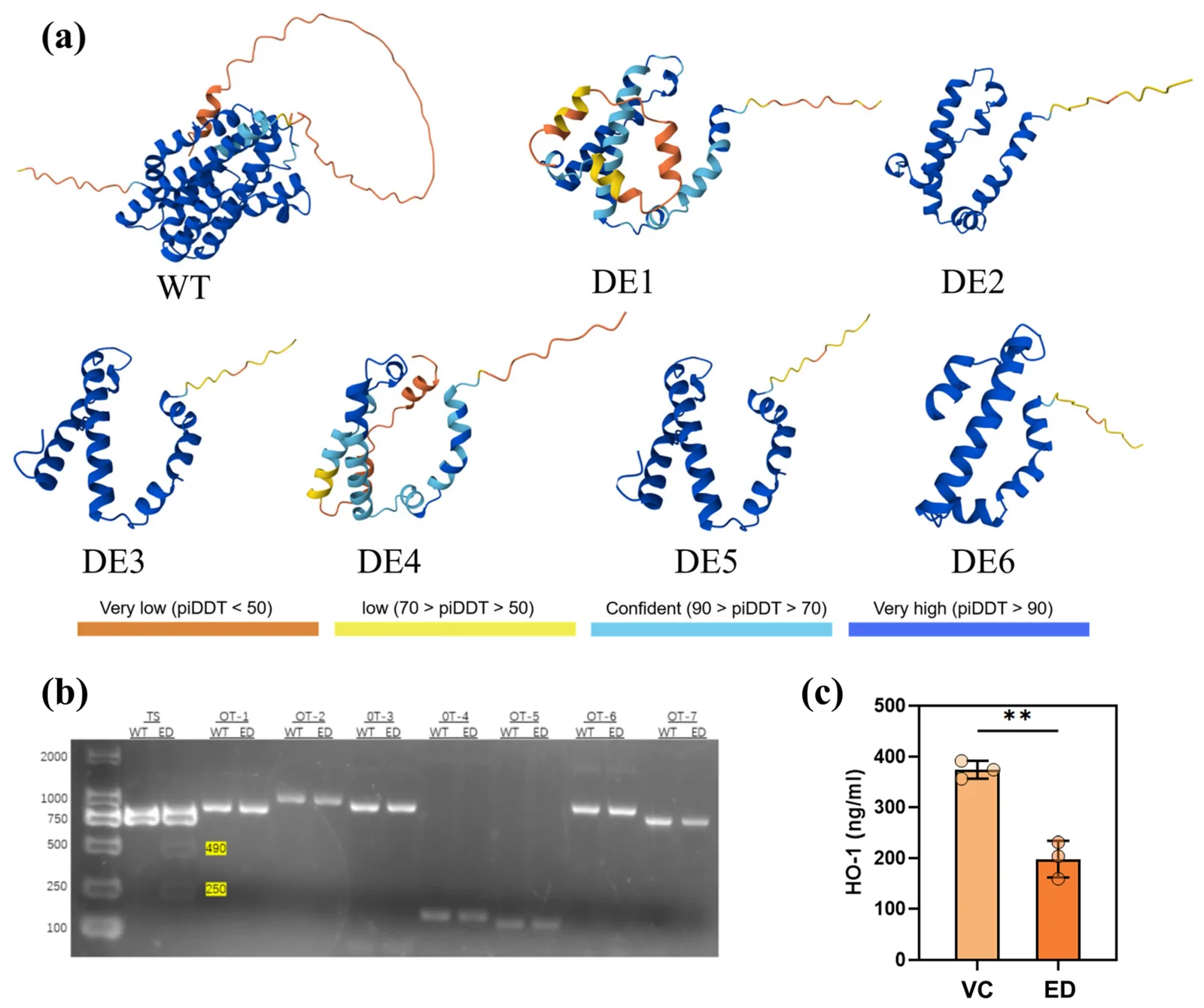
Predicted structural consequences of the *HMOX1* deletion alleles, targeted off-target assessment, and HO-1 protein abundance in *HMOX1*-edited LMH cells. (**a**) AlphaFold 3-predicted monomeric structures of full-length WT HO-1 and the inferred translation products encoded by the six distinct deletion alleles, DE1–DE6, identified in the initial sgRNA2 screening population. Models are colored according to the predicted local distance difference test score (pLDDT): very low confidence, pLDDT < 50; low confidence, 50 ≤ pLDDT < 70; confident, 70 ≤ pLDDT < 90; and very high confidence, pLDDT ≥ 90. The DE1–DE6 models were generated from reconstructed mutant coding sequences based on Sanger-confirmed deletion alleles. (**b**) T7 endonuclease I assay of the intended *HMOX1* target site (TS) and seven predicted candidate off-target loci (OT-1–OT-7). Cleavage products of approximately 490 and 250 bp were detected at the intended target site in ED cells, whereas no evident cleavage products were detected at the seven examined candidate off-target loci under the assay conditions used. (**c**) HO-1 protein abundance in vector-control (VC) and *HMOX1*-edited (ED) LMH cells measured using a chicken-specific HO-1 ELISA kit. Bars represent the mean ± SD of three independently transfected and GFP-sorted biological replicates, and individual points represent biological replicates. Statistical significance was assessed using an unpaired two-tailed Student’s *t*-test. ** *p* < 0.01.

To visualize the potential structural consequences of these sequence alterations, the full-length WT HO-1 sequence and the inferred translation products of DE1–DE6 were subjected to AlphaFold 3 monomeric structure prediction. The WT model represented the full-length 296-amino-acid HO-1 protein, whereas the six edited alleles yielded markedly shortened predicted products with loss of the native catalytic and C-terminal regions (Figure 1a). Model-confidence parameters, including pLDDT, pTM, and ranking scores, are provided in Table S1.

Potential editing at seven candidate off-target loci predicted by Cas-OFFinder was examined using T7 endonuclease I assays. Detectable cleavage products were observed at the intended *HMOX1* target site, whereas no evident cleavage products were detected at OT-1 to OT-7 under the assay conditions used (Figure 1b). These results indicate that no readily detectable editing was identified at the seven examined candidate loci but do not establish general or genome-wide editing specificity (Figure 1b).

HO-1 protein abundance was subsequently measured in independently generated VC and ED cell preparations. ELISA analysis showed that HO-1 abundance was reduced by approximately 47.0% in *HMOX1*-edited (ED) cells relative to vector-control (VC) cells (Figure 1c; *p* < 0.01; *n* = 3). The lower magnitude of the reduction in HO-1 abundance relative to the clone-level Indel frequency was consistent with the heterogeneous composition of the edited cell populations, including residual unedited alleles and multiple edited alleles with potentially different transcriptional and protein-level consequences.

Together, these results show that sgRNA2 efficiently generated heterogeneous *HMOX1*-edited LMH cell populations containing multiple frameshift deletion alleles and that independently generated ED populations exhibited substantially reduced HO-1 protein abundance.

### 2.2. HMOX1 Editing Alters Heme Metabolism, Cellular Redox Status, and CCK-8 Activity

**Figure 2:**
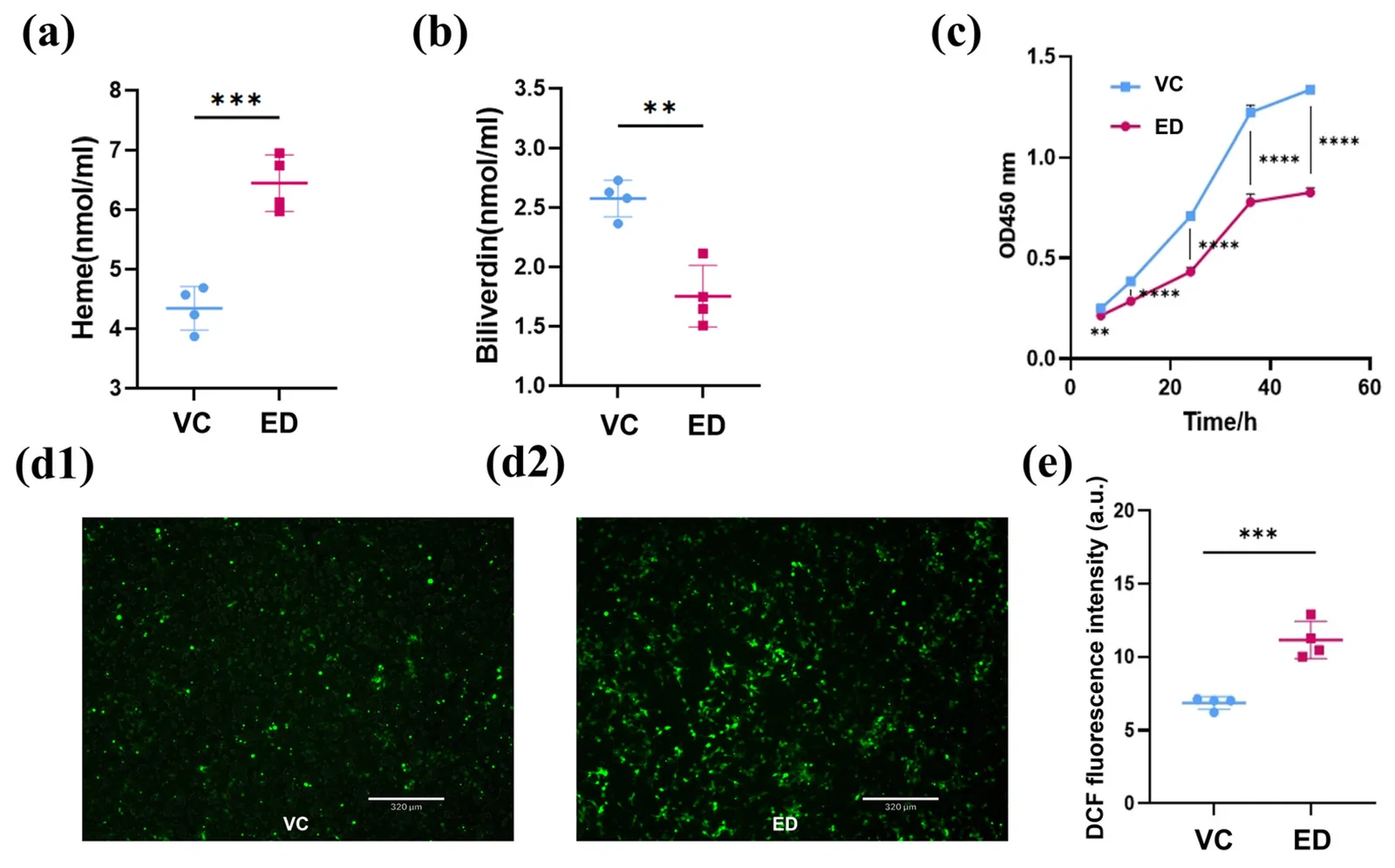
*HMOX1* editing alters the heme–biliverdin balance, CCK-8 reductive/metabolic activity, and intracellular oxidation-sensitive fluorescence in LMH cells. (**a**) Intracellular heme concentrations in vector-control (VC) and *HMOX1*-edited (ED) cells. (**b**) Intracellular biliverdin concentrations in VC and ED cells. (**c**) CCK-8 absorbance measured at 6, 12, 24, 36, and 48 h after equal-density seeding. OD_450_ values reflect the cellular reduction in the CCK-8 substrate and are used as an indicator of metabolic/reductive activity. (**d1**,**d2**) Representative confocal fluorescence images of DCFH-DA-stained VC and ED cells, respectively. Scale bars, 320 μm. (**e**) Quantification of DCF oxidation-sensitive fluorescence. Five randomly selected microscopic fields were analyzed for each biological replicate, and the mean field value was used as the value for that replicate; individual fields were not treated as independent replicates. Data are presented as the mean ± SD of four independently transfected and GFP-sorted biological replicates per group; individual symbols in (**a**,**b**,**e**) represent biological replicates. ** *p* < 0.01, *** *p* < 0.001, and **** *p* < 0.0001.

We next examined the cellular phenotypes associated with *HMOX1* editing. Compared with VC cells, ED cells showed a significant increase in intracellular heme levels, corresponding to an approximate elevation of 48.5% (Figure 2a; *p* < 0.001; *n* = 4). In contrast, intracellular biliverdin levels were reduced by approximately 31.9% in ED cells (Figure 2b; *p* < 0.01; *n* = 4). The reciprocal increase in heme and decrease in biliverdin was consistent with reduced HO-1-dependent heme degradation in the edited cell populations.

CCK-8 assays showed lower OD_450_ values in ED cells than in VC cells over the examined period from 6 to 48 h (Figure 2c; *n* = 4). Although OD_450_ values increased progressively in both groups, the difference between VC and ED cells became more pronounced after 24 h, indicating reduced cellular reductive/metabolic activity in the *HMOX1*-edited cells.

In parallel, DCFH-DA staining revealed stronger fluorescence signals in ED cells than in VC cells (Figure 2(d1),(d2)). Quantitative analysis confirmed a significant increase in intracellular oxidation-sensitive fluorescence in ED cells (Figure 2e; *n* = 4). For each biological replicate, fluorescence values from multiple randomly selected microscopic fields were averaged before statistical analysis.

Collectively, these results show that *HMOX1*-edited LMH cells exhibit an altered heme–biliverdin balance, an elevated intracellular oxidative state, and reduced CCK-8 absorbance.

### 2.3. HMOX1 Editing Is Associated with Coordinated Remodeling of Basal Immune, Stress, and Cell-Cycle-Related Transcriptional Programs in LMH Cells

**Figure 3:**
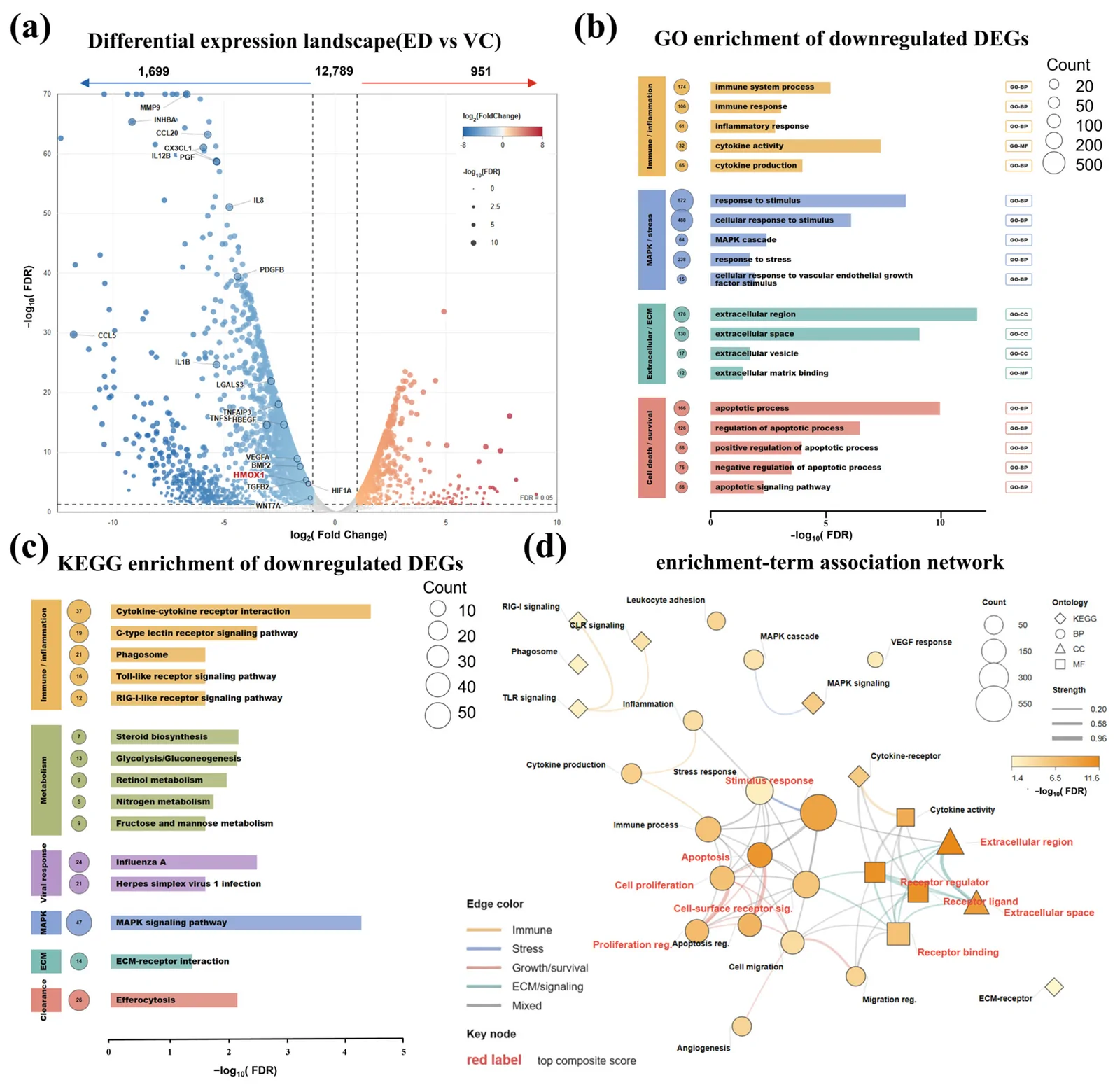
Differential expression and functional enrichment profiles of *HMOX1*-edited LMH cells. (**a**) Volcano plot showing differential gene expression between *HMOX1*-edited (ED) and vector-control (VC) cells. Each point represents one gene. Blue and orange points indicate significantly downregulated and upregulated genes in ED cells, respectively, whereas gray points indicate genes that did not meet the differential-expression criteria. Differentially expressed genes were defined by absolute log_2_(Fold change) ≥ 1 and false discovery rate (FDR) < 0.05. A total of 1699 genes were downregulated and 951 genes were upregulated, whereas 12,789 genes were not classified as differentially expressed. Selected genes are labeled. (**b**) Representative Gene Ontology (GO) terms enriched among downregulated differentially expressed genes. Enriched terms were grouped into immune/inflammation-, MAPK/stress, extracellular matrix/extracellular region-, and cell death/survival-related functional categories. Bar length represents −log_10_(FDR), circle size represents the number of mapped genes, and labels on the right indicate the corresponding GO category: biological process (BP), cellular component (CC), or molecular function (MF). (**c**) Representative KEGG pathways enriched among downregulated differentially expressed genes. Pathways were grouped into immune/inflammation, metabolism, viral response, MAPK signaling, extracellular matrix, and clearance-related categories. Bar length represents −log_10_(FDR), and circle size represents the number of mapped genes. (**d**) Enrichment-term association network constructed from significantly enriched GO and KEGG terms identified among downregulated genes. Connections between terms represent shared query genes, and edge strength reflects the degree of gene-set overlap. Node shape indicates ontology category, including KEGG, GO biological process, GO cellular component, and GO molecular function. Node size represents the number of mapped genes, and node fill represents enrichment significance expressed as −log_10_(FDR). Terms highlighted with red labels indicate key highly connected nodes. Functional categories of network connections are indicated by edge colors.

To characterize the transcriptional changes associated with reduced HO-1 abundance, RNA sequencing was performed in independently prepared VC and ED cell populations. Principal component analysis showed clear separation between the two groups, indicating distinct transcriptional profiles following *HMOX1* editing (Figure S2). Differential expression analysis identified 2650 differentially expressed genes (DEGs), including 951 upregulated and 1699 downregulated genes in ED cells relative to VC cells (|log_2_(Fold change)| ≥ 1 and FDR < 0.05; Figure 3a). Thus, the transcriptional response was characterized by a predominance of downregulated genes.

Functional enrichment analysis showed that downregulated genes were mainly associated with immune and inflammatory regulation, cytokine-mediated signaling, responses to stimuli, extracellular communication, and cell-fate regulation (Figure 3b). KEGG enrichment analysis further identified cytokine–cytokine receptor interaction, Toll-like receptor signaling, C-type lectin receptor signaling, MAPK signaling, focal adhesion, and ECM–receptor interaction among the major downregulated pathways (Figure 3c). In contrast, genes upregulated in ED cells were primarily enriched in DNA replication and cell cycle-related processes (Figure S3). These findings revealed coordinated remodeling of basal immune, stress, extracellular signaling, and cell-cycle-associated transcriptional programs in *HMOX1*-edited LMH cells.

To examine the functional relationships among the enriched categories, an enrichment-term association network was constructed based on genes shared among representative GO and KEGG terms identified from the downregulated DEG set. The network contained 29 enriched terms and 60 term-to-term associations. Highly connected terms included receptor ligand activity, signaling receptor regulator activity, cell surface receptor signaling pathway, response to stimulus, cell population proliferation, apoptotic process, signaling receptor binding, extracellular region, and extracellular space (Figure 3d). These terms formed an interconnected functional landscape involving extracellular signaling, immune and stress-related processes, cell proliferation, apoptosis, migration, and extracellular matrix organization.

**Figure 4:**
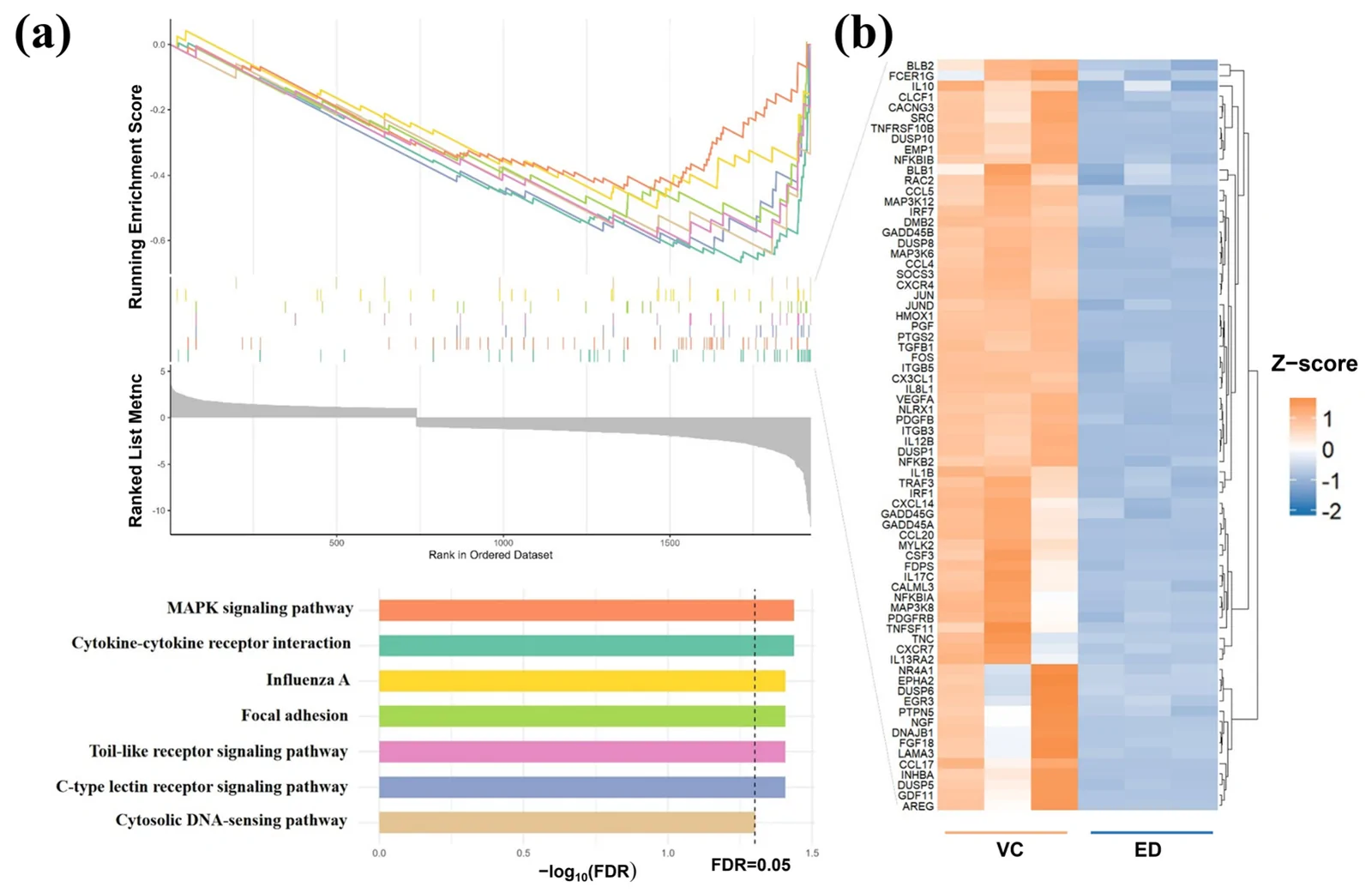
Gene set enrichment analysis and expression patterns of core enrichment genes in *HMOX1*-edited LMH cells. (**a**) Gene set enrichment analysis (GSEA) of the ED-versus-VC transcriptomic comparison showing selected KEGG pathways negatively enriched in *HMOX1*-edited (ED) cells. Running enrichment-score curves are shown for the MAPK signaling pathway, cytokine–cytokine receptor interaction, influenza A, focal adhesion, Toll-like receptor signaling pathway, C-type lectin receptor signaling pathway, and cytosolic DNA-sensing pathway. Vertical marks indicate the positions of pathway-associated genes in the ranked gene list. The lower bar plot shows the statistical significance of the selected pathways as −log_10_(FDR), and the dashed vertical line indicates the FDR = 0.05 threshold. (**b**) Heatmap showing the expression patterns of core enrichment genes contributing to the significantly enriched pathways shown in panel (**a**). Rows represent genes, and columns represent independently transfected and GFP-sorted biological replicates from the vector-control (VC) and ED groups (*n* = 3 per group). Expression values were log_2_(FPKM + 1) transformed and standardized by gene across all samples. The color scale represents row-wise Z-scores, with orange and blue indicating relatively high and low expression, respectively.

GSEA was subsequently performed using the ranked transcriptomic dataset. Consistent with the DEG-based enrichment results, several immune-, stress-, and extracellular signaling-associated pathways showed negative enrichment in ED cells, including MAPK signaling, cytokine–cytokine receptor interaction, Toll-like receptor signaling, C-type lectin receptor signaling, focal adhesion, and the cytosolic DNA-sensing pathway (Figure 4a). Core genes contributing to these negatively enriched pathways, including *IL1B*, *IRF1*, *JUN*, and *NFKBIA*, also showed lower expression in ED cells in the RNA-seq dataset (Figure 4b).

RT-qPCR analysis was performed to assess selected expression patterns identified by RNA sequencing. The relative mRNA levels of *IRF1*, *JUN*, *NFKBIA*, and *PTGS2* were significantly lower in ED cells than in VC cells, whereas the DNA replication and cell cycle-associated genes *PCNA* and *MCM2* were significantly upregulated in ED cells (Figure S4). *JUN* and *PTGS2* showed a significant difference at *p* < 0.01, whereas *IRF1*, *NFKBIA*, *PCNA*, and *MCM2* differed at *p* < 0.05. These RT-qPCR results were consistent with the direction of expression changes observed in the RNA-seq dataset.

*IL1B* transcripts were detected at low abundance in VC cells, with a mean Ct value of 35.92, but were not detected in ED cells under the same amplification conditions. Because relative expression could not be calculated using the conventional 2^−ΔΔCt^ method when no Ct value was obtained in the ED group, *IL1B* was reported qualitatively and was not included in the statistical comparison of relative expression levels. The loss of detectable *IL1B* amplification in ED cells was nevertheless consistent with its marked downregulation in the RNA-seq dataset.

**Figure 5:**
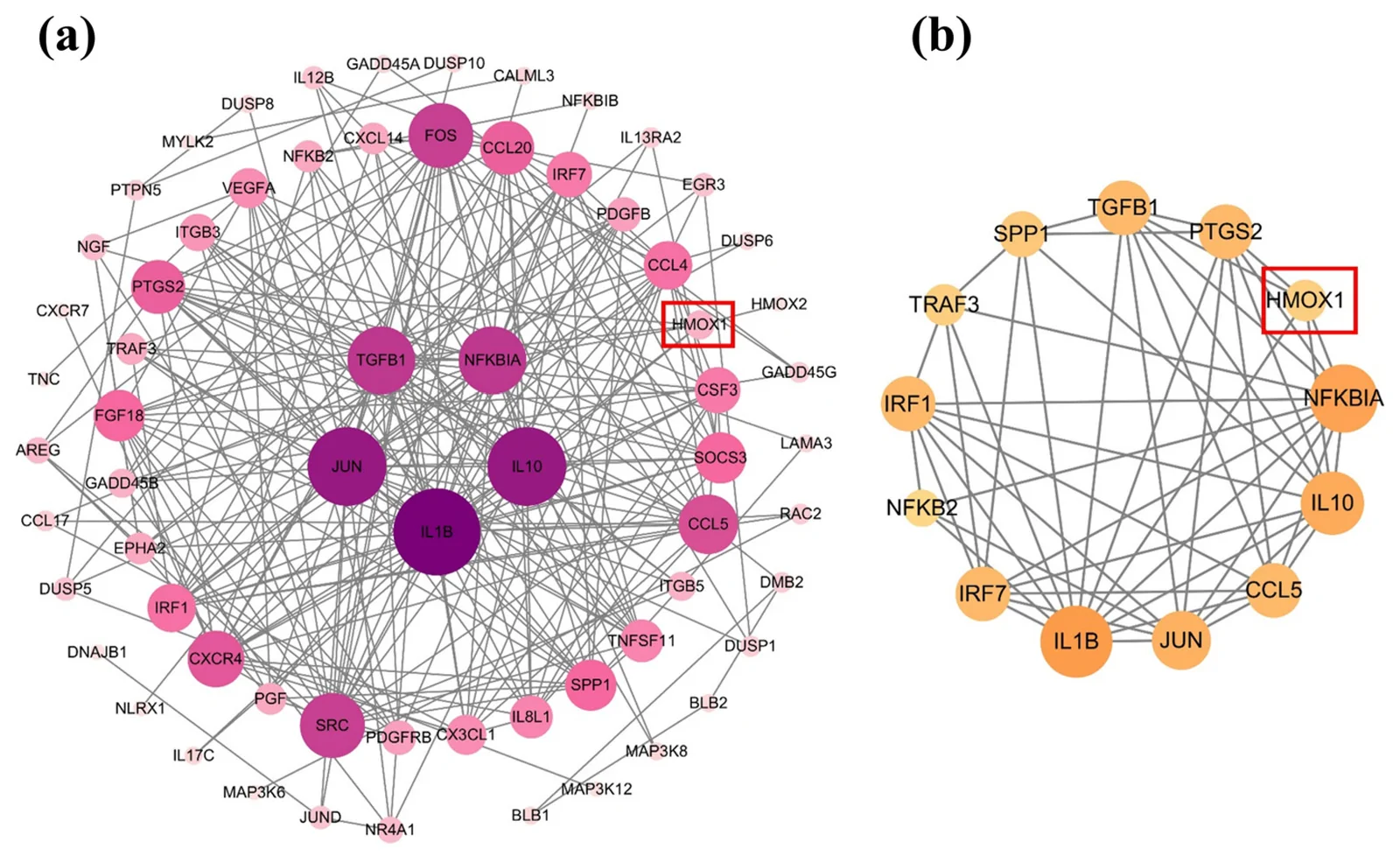
Network analysis links *HMOX1* with immune and stress-related regulatory genes. (**a**) STRING functional-association network integrating *HMOX1* with core genes from GSEA-enriched KEGG pathways. (**b**) MCODE-derived subnetwork connecting *HMOX1* with representative immune and stress regulators, including *NFKBIA*, *IL10*, *CCL5*, *IRF7*, *IL1B*, *JUN*, *IRF1*, *TGFB1*, and *PTGS2*. The red boxes indicate *HMOX1*.

To further characterize the functional context of the affected genes, *HMOX1* and selected core genes from the enriched pathways were incorporated into a STRING functional association network. The resulting network contained a highly interconnected module involving *IL1B*, *JUN*, *NFKBIA*, *TGFB1*, *IL10*, *SRC*, *PTGS2*, and *CCL5* (Figure 5a). MCODE analysis further identified a densely connected subnetwork containing *HMOX1* and several immune- and stress-associated genes, including *NFKBIA*, *IL10*, *CCL5*, *IRF7*, *IL1B*, *JUN*, *IRF1*, *TGFB1*, and *PTGS2* (Figure 5b).

Together, the transcriptome-wide analyses and RT-qPCR results show that *HMOX1* editing in LMH cells is associated with coordinated downregulation of basal immune-, stress-, and extracellular signaling-associated genes, accompanied by increased expression of selected DNA replication and cell cycle-related genes.

## 3. Discussion

HO-1 catalyzes the rate-limiting step of heme degradation, producing biliverdin, carbon monoxide, and ferrous iron, and plays an important role in cellular redox regulation and stress-associated signaling [19,20]. In mammalian systems, HO-1 is widely recognized for its cytoprotective and immunomodulatory functions [21,22]. In the present study, heterogeneous *HMOX1* editing in LMH cells reduced HO-1 protein abundance and was accompanied by intracellular heme accumulation, reduced biliverdin levels, increased oxidation-sensitive fluorescence, and lower CCK-8 reductive/metabolic activity. Transcriptomic analysis further revealed coordinated remodeling of basal immune, cytokine, MAPK/stress, extracellular signaling, and cell-cycle-associated expression programs. These findings indicate that reduced HO-1 abundance in chicken hepatic cells is associated with broader metabolic and transcriptional consequences beyond heme degradation alone.

The difference between the clone-level Indel frequency and the magnitude of HO-1 protein reduction is consistent with the heterogeneous composition of the edited cell populations. In the initial sgRNA2 screening, 29 of 42 TA clones contained Indels, whereas 13 retained the WT target sequence. By comparison, HO-1 protein abundance was reduced by approximately 47% in independently generated ED samples. This difference is biologically plausible because clone-level editing frequency does not directly correspond to total cellular protein abundance. Residual unedited alleles, variation among edited alleles, and differences in transcript or protein stability may all contribute to the population-average HO-1 level. Accordingly, the ED group represents a heterogeneous *HMOX1*-edited cell population with reduced average HO-1 abundance rather than a uniform single-genotype model.

The metabolic phenotype was consistent with attenuation of HO-1-mediated heme turnover. HO-1 converts heme into biliverdin, carbon monoxide, and ferrous iron [1]; therefore, the simultaneous accumulation of intracellular heme and reduction in biliverdin support altered heme degradation in the edited cells. This reduction in biliverdin may be particularly relevant in the avian context. Unlike mammals, birds have relatively limited conversion of biliverdin to bilirubin, and biliverdin itself represents a major avian bile pigment and heme-derived metabolite [9,10]. Thus, reduced biliverdin may reflect not only lower heme-catabolic output but also a decrease in an avian-relevant antioxidant-associated metabolite [23]. More broadly, the limited conversion of biliverdin to bilirubin makes the avian system a valuable natural model for investigating the biological functions of HO-1-derived biliverdin while minimizing potential confounding effects from downstream bilirubin metabolism.

The altered heme–biliverdin balance also provides a plausible biochemical context for the increased oxidation-sensitive fluorescence observed in ED cells. Excess free heme can promote oxidative reactions, whereas biliverdin has been associated with antioxidant activity [23,24,25]. Their reciprocal changes may therefore contribute to the altered intracellular redox state. Although the present data do not resolve the relative contribution of each metabolite, the coordinated increase in heme, reduction in biliverdin, and elevation of DCFH-DA fluorescence support disruption of redox homeostasis following *HMOX1* editing.

Notably, ED cells showed coordinated enrichment of both DNA replication/cell-cycle programs and DNA repair/replication-fork maintenance programs. The increased expression of the *MCM2–7*, *ORC* and *GINS* complexes, together with *CDC45*, *PCNA* and *CCNE1/2*, indicates enhanced transcriptional preparation for replication licensing and initiation [26,27,28]. The concurrent increase in the S-phase-regulated repressors *E2F7* and E2F8 may additionally reflect feedback regulation of this replication-associated program [29]. Upregulation of *BRCA1*, *BRCA2*, *PALB2* and *RAD51* suggests an increased requirement for homologous recombination and stalled-fork protection [30,31,32,33], whereas *ATAD5*, *WRN*, *DNA2* and *RIF1* are involved in *PCNA* regulation, fork restart and controlled processing of reversed replication forks [34,35,36]. This coordinated transcriptional pattern is therefore consistent with a compensatory replication-stress response, in which cells reinforce both replication and genome-maintenance mechanisms when fork progression is challenged [37,38].

This model provides a mechanistic explanation for the apparent difference between the transcriptomic and CCK-8 results. Increased expression of replication-associated genes may represent an attempt to maintain or restart DNA replication rather than a net increase in productive cell proliferation. Notably, HO-1 deficiency and excess heme have been linked experimentally to oxidative DNA damage, replication-fork stalling and replication stress [25,39], providing a mechanistic connection between the heme accumulation and increased oxidation-sensitive fluorescence observed in ED cells and the transcriptional activation of fork-maintenance programs. The initial Cas9-induced double-strand break may also have contributed to the DNA-repair-associated response [40,41]. Meanwhile, because WST-8 reduction is strongly influenced by cellular glucose metabolism and reductive capacity, the lower CCK-8 signal may reflect impaired metabolic output despite activation of replication-associated transcription [42]. Together, these findings suggest a partial uncoupling between replication-associated transcription and cellular reductive/metabolic activity following *HMOX1* editing.

A prominent transcriptomic feature of *HMOX1*-edited LMH cells was the coordinated repression of genes associated with immune regulation, cytokine signaling, MAPK-related processes, extracellular communication, and responses to stimuli. Independent RT-qPCR analysis reproduced the principal expression trends detected by RNA sequencing, including reduced expression of *IRF1*, *JUN*, *NFKBIA*, and *PTGS2* and increased expression of *PCNA* and *MCM2*. This agreement supports the reproducibility of the major transcriptional patterns in an independent biological sample set.

The repression of immune and stress-associated genes was notable because increased intracellular oxidation is often associated with activation of stress-responsive transcription. In the present study, however, elevated oxidation-sensitive fluorescence was accompanied by reduced basal expression of multiple cytokine and stress-associated genes. This suggests that reduced HO-1 abundance may be associated with an altered regulatory state in which oxidative imbalance does not lead to uniform activation of canonical stress-response pathways.

Previous studies have shown that chicken *HMOX1* is inducible by stress-related stimuli such as sodium arsenite and cobalt chloride, supporting its involvement in avian cellular stress regulation [14,15]. In mammalian HO-1-deficient models, reduced HO-1 function has also been linked to oxidative injury, abnormal heme and iron handling, endothelial dysfunction, and altered inflammatory signaling [43,44,45]. Together with these observations, the present results suggest that HO-1 may contribute not only to the enzymatic removal of heme but also to the maintenance of basal stress-associated transcriptional homeostasis in avian hepatic cells.

Among the downregulated genes, *IRF1* is involved in interferon-associated transcription, whereas *JUN* contributes to AP-1-mediated stress and mitogenic signaling [44,46]. *PTGS2* participates in inflammatory lipid-mediator synthesis, and its reduction is consistent with the broader suppression of inflammatory-response genes. *NFKBIA* encodes an inhibitor and feedback regulator of NF-κB signaling. Although reduced *NFKBIA* expression alone does not define the direction of NF-κB activity, its alteration together with widespread repression of cytokine and receptor-signaling genes indicates disturbance of the basal inflammatory regulatory network.

The enrichment-term association network placed these changes within a broader functional framework. Downregulated GO and KEGG terms were linked mainly through shared genes involved in receptor–ligand activity, cell-surface receptor signaling, extracellular regions, responses to stimuli, proliferation, and apoptotic regulation. This pattern indicates coordinated remodeling of extracellular communication and stimulus-associated biological functions rather than isolated changes in individual pathways.

Consistently, STRING functional-association and MCODE analyses identified a network containing *HMOX1* together with *IL1B*, *JUN*, *NFKBIA*, *TGFB1*, *IL10*, *CCL5*, *IRF7*, *PTGS2*, and other genes involved in cytokine and stress-associated regulation [47,48,49,50,51,52]. These functional associations support the existence of a broader *HMOX1*-associated regulatory module linking heme metabolism, redox balance, and inflammatory signaling. HO-1-derived metabolites, including carbon monoxide and biliverdin/bilirubin-pathway intermediates, are also known to influence redox- and inflammation-related signaling in a context-dependent manner. Altered production of these metabolites may therefore contribute to the transcriptional remodeling observed in *HMOX1*-edited LMH cells.

The present findings should be interpreted in the context of several experimental considerations. The ED samples were heterogeneous GFP-positive cell populations containing multiple edited alleles and residual unedited alleles, so the observed phenotypes represent population-average effects. In addition, HO enzymatic activity was not measured directly, and the DCFH-DA and CCK-8 assays provide general indicators of cellular oxidation-sensitive fluorescence and reductive/metabolic activity, respectively. The transcriptomic measurements were obtained under basal culture conditions and therefore describe an altered basal transcriptional state rather than the magnitude of the response to a defined external stimulus. Future studies using genotype-resolved clones, additional sgRNAs, metabolic rescue, direct HO activity measurements, and defined stress challenges would help clarify the mechanistic relationships among *HMOX1* editing, heme metabolism, redox balance, and transcriptional regulation.

Taken together, heterogeneous *HMOX1* editing and the associated reduction in HO-1 protein abundance were accompanied by disruption of the heme–biliverdin metabolic axis, increased intracellular oxidation-sensitive fluorescence, reduced CCK-8 reductive/metabolic activity, and coordinated remodeling of basal immune, stress, extracellular signaling, and cell-cycle-associated transcriptional programs in LMH cells. These findings support a role for HO-1 in linking heme metabolism with redox and transcriptional homeostasis in LMH cells.

## 4. Materials and Methods

### 4.1. Cell Culture, Transfection, and Plasmid Construction

LMH cells, a chicken hepatocellular carcinoma-derived cell line, were cultured in DMEM/F-12 medium (Solarbio, Beijing, China; Cat. No. D6570) supplemented with 10% fetal bovine serum (FBS; Thermo Fisher Scientific, Waltham, MA, USA; Cat. No. 26010074) at 37 °C in a humidified atmosphere containing 5% CO_2_. LMH cells were used as an avian hepatic cell model for *HMOX1* functional analysis because of their hepatic origin and established use in chicken liver-related studies [53,54,55].

Three sgRNAs, designated sgRNA1, sgRNA2, and sgRNA3, targeting exon 3 of chicken *HMOX1* were designed based on the *Gallus gallus* GRCg7b reference genome sequence (RefSeq accession No. NC_052532.1). Candidate sgRNAs were selected according to standard SpCas9 design criteria, including the presence of an NGG protospacer-adjacent motif, a GC content of 40–60%, and a predicted off-target risk score ≤ 0.3 [56]. The sgRNA sequences are provided in Table S2.

Complementary oligonucleotides were synthesized by BGI (Shenzhen, China), annealed, and separately ligated into the BbsI site of the PX458 plasmid (pSpCas9(BB)-2A-GFP; Addgene plasmid #48138, Watertown, MA, USA), which expresses SpCas9 and GFP (Figure S5). Plasmid construction was performed using BbsI-HF restriction endonuclease (New England Biolabs, Ipswich, MA, USA; Cat. No. R3539) and T4 DNA ligase (New England Biolabs, Ipswich, MA, USA; Cat. No. M0569S). The ligation products were transformed into DH5α competent cells (Genesand Biotech, Beijing, China; Cat. No. SCC12), and positive clones were verified by Sanger sequencing.

For the initial sgRNA screening, LMH cells at approximately 70% confluence were transfected in a single experimental batch without biological replication with PX458-sgRNA1, PX458-sgRNA2, PX458-sgRNA3, or empty PX458 vector using Lipofectamine 3000 (Thermo Fisher Scientific, Carlsbad, CA, USA; Cat. No. L3000015) according to the manufacturer’s instructions. At 48 h post-transfection, GFP-positive cells were isolated by fluorescence-activated cell sorting (FACS) and used for genome-editing validation. This initial screening was conducted exclusively to compare clone-level Indel proportions among the three sgRNAs and was not used for statistical inference regarding cellular phenotypes.

Based on the initial screening results, sgRNA2 was selected to generate the heterogeneous *HMOX1*-edited cell populations used in subsequent experiments. These cells are hereafter referred to as the edited group (ED). Cells transfected with empty PX458 are hereafter referred to as the vector-control group (VC). The VC cells received PX458 lacking an sgRNA sequence and underwent the same transfection, GFP-positive cell sorting, post-sorting recovery, passaging, seeding, and sampling procedures as the ED cells. The term “WT” is used only to denote the reference *HMOX1* sequence or an unedited allele and is not used to designate the cellular control group.

Each biological replicate used in downstream experiments was derived from an independently cultured LMH cell preparation and independently underwent plasmid transfection, GFP-positive cell sorting, and post-sorting recovery. Cells from different biological replicates were not pooled. Technical wells or microscopic fields derived from the same independently prepared cell sample were not considered independent biological replicates.

Experimental series A consisted of three independently prepared sgRNA2-edited cell samples and three independently prepared empty-vector control samples. These samples were used for RNA sequencing and HO-1 protein quantification.

Experimental series B consisted of four independently prepared sgRNA2-edited cell samples and four independently prepared empty-vector control samples. These samples were used for intracellular heme, biliverdin, CCK-8, and DCFH-DA assays. Untreated parallel cultures derived from three independently prepared samples per group within experimental series B were reserved for RNA extraction and RT-qPCR validation. These parallel cultures were not exposed to CCK-8, DCFH-DA, heme-assay, biliverdin-assay, or other functional-assay reagents before RNA collection. Therefore, the RT-qPCR sample set comprised three independent biological replicates per group and was independent of the samples used for RNA sequencing.

RNA-seq and RT-qPCR samples were collected using the same predefined sampling strategy. Within each experimental batch, VC and ED cells were harvested in parallel at the same elapsed time after seeding. At the time of collection, cultures in both groups had reached greater than 70% confluence. The experimental series, independent transfection identifier, independent sorting identifier, post-sorting culture duration, passage number, sampling time, confluence at collection, and experimental application of each sample are provided in Table S3.

### 4.2. Genome Editing Validation and Off-Target Screening

To evaluate the editing efficiencies of the three sgRNAs, genomic DNA was extracted from GFP-positive cells isolated after the initial single-batch transfection using a genomic DNA extraction kit (TIANGEN Biotech, Beijing, China; Cat. No. DP304). The *HMOX1* target region was amplified by PCR using primers YW1 and YW2. PCR products were cloned using the TA pTOPO001 Blunt Simple Cloning Kit (Genesand Biotech, Beijing, China; Cat. No. TA pTOPO001), and positive clones were sequenced using M13 universal primers. Primer sequences are provided in Table S2.

Sequencing chromatograms were aligned against the reference *HMOX1* sequence and analyzed using SnapGene (v4.3). Only clones with unambiguous sequencing chromatograms spanning the sgRNA target region were included in the analysis. Alleles identical to the reference *HMOX1* target sequence were classified as WT. Indels preserving the original *HMOX1* coding frame were classified as in-frame alleles, whereas Indels altering the coding frame were classified as frameshift alleles. The number and proportion of each individual allele and the combined frequencies of WT, in-frame, and frameshift alleles were calculated relative to the total number of valid sequenced clones.

The clone-level Indel frequency was calculated as follows:

$$Indel\;frequnency\left(\%\right)=\;\frac{number\;of\;mutant\;clones}{total\;number\;of\;valid\;sequenced\;clones}\;\times 100\%$$

Based on this initial screening, sgRNA2, which produced the highest clone-level Indel frequency, was selected for the generation of *HMOX1*-edited cell populations in all subsequent experimental batches.

Potential off-target sites of sgRNA2 were predicted using Cas-OFFinder (accessed on 18 June 2025) [57]. The seven top-ranked candidate off-target loci, designated OT-1 to OT-7, were selected for targeted examination. Their genomic locations, candidate sequences, bulge sizes, and mismatch numbers are provided in Table S4. Site-specific primers used to amplify the intended target region and the seven candidate off-target loci are provided in Table S5.

The corresponding genomic regions were amplified by PCR, and the resulting products were subjected to T7 endonuclease I cleavage assays using T7 Endonuclease I (New England Biolabs, Ipswich, MA, USA; Cat. No. E3321S). This assay was used as a targeted screen for readily detectable editing at the examined candidate loci. Because T7E1 has limited sensitivity for low-frequency editing events, the absence of evident cleavage products was not interpreted as evidence of genome-wide editing specificity.

### 4.3. Prediction of the Coding and Structural Consequences of HMOX1 Editing and Quantification of HO-1 Protein Abundance

To evaluate the potential effects of *HMOX1* editing on HO-1 protein integrity, the sgRNA target sites were mapped to the chicken HO-1 amino acid sequence. According to UniProt annotation [58], the catalytic residue Asp143 and the C-terminal structural region spanning amino acids 231–264 are located downstream of the sgRNA target sites. The three sgRNAs targeted coding regions corresponding to amino acids 92–99, 105–111, and 122–129 of HO-1, respectively. Frameshift mutations introduced at these positions were therefore expected to alter the downstream amino acid sequence and potentially affect the native catalytic and C-terminal regions of HO-1.

To predict the coding consequences of sgRNA2-mediated editing, PCR products spanning the *HMOX1* target region from the initial screening batch were subjected to TA cloning and Sanger sequencing. Six high-confidence deletion alleles, designated DE1–DE6, were selected for further analysis. Each Sanger-confirmed deletion was individually incorporated into the full-length coding sequence of the selected chicken *HMOX1* transcript (accession No. XM_046921508.1) to reconstruct the corresponding mutant coding sequence.

The reconstructed mutant coding sequences were translated in silico from the native *HMOX1* initiation codon in the original coding frame using SnapGene (v4.3). The resulting inferred translation products were aligned with the WT HO-1 amino acid sequence to identify retained WT amino acid regions, frameshift-derived non-native sequences, premature termination codons, and predicted translation-product lengths. Particular attention was given to the retention or loss of the native catalytic residue Asp143 and the C-terminal region spanning amino acids 231–264.

To visualize the potential structural consequences of the sequence alterations, the full-length WT HO-1 amino acid sequence and the inferred translation products of DE1–DE6 were separately submitted to AlphaFold 3 (https://alphafoldserver.com/, accessed on 15 July 2026) for monomeric protein-structure prediction. Predictions were performed using the AlphaFold 3 server in monomer mode with default settings. No user-supplied interacting protein chains, nucleic acids, ligands, or other molecular components were included.

Predicted structures were downloaded in PDB or mmCIF format. Model-confidence parameters, including the predicted local distance difference test score (pLDDT), predicted template modeling score (pTM), and ranking score, were recorded for each model and are provided in Table S1. The structural models represent hypothetical translation products inferred from edited nucleotide sequences and were used to visualize potential structural consequences of the identified sequence alterations.

For experimental quantification, HO-1 protein abundance was measured using experimental series A, comprising three independently transfected and sorted biological replicates per group. A chicken-specific HO-1 ELISA kit (Shanghai Coibo Biotechnology Co., Ltd., Shanghai, China; Cat. No. CB10488-Ch) was used according to the manufacturer’s instructions.

Briefly, equal numbers of VC and ED LMH cells were collected, resuspended in PBS, and lysed by three repeated freeze–thaw cycles. After centrifugation to remove cellular debris, the supernatants were collected and diluted five-fold before measurement. Absorbance was measured at 450 nm, and HO-1 concentrations were calculated from the standard curve and corrected for the dilution factor.

### 4.4. Functional Assays

All functional assays were performed using experimental series B, comprising four independently transfected and sorted biological replicates per group.

#### 4.4.1. Intracellular Heme Measurement

Intracellular heme levels were determined using a Heme Assay Kit (FineTest, Wuhan, China; Cat. No. EU2610), which is based on heme-catalyzed oxidation of 3,3′,5,5′-tetramethylbenzidine (TMB).

Equal numbers of VC and ED LMH cells were collected, washed with ice-cold physiological saline, and lysed in equal volumes of 0.9% NaCl containing protease inhibitors. The lysates were sonicated on ice and centrifuged at 10,000 rpm for 10 min at 2–8 °C.

The resulting supernatants were incubated with TMB substrate at 37 °C for 20 min in the dark. Serial heme standards ranging from 15.625 to 1000 pmol/mL were processed in parallel. After termination of the reaction, absorbance was measured at 450 nm. Heme concentrations were calculated from the standard curve and expressed as nmol/mL of cell lysate after unit conversion.

#### 4.4.2. Biliverdin Measurement

For biliverdin quantification, equal numbers of VC and ED LMH cells were lysed and centrifuged using the same procedure as described for the heme assay. The cleared supernatants were mixed with extraction buffer and incubated at room temperature for 12 h in the dark. A standard curve was generated using serially diluted biliverdin standards, and biliverdin concentrations were determined by measuring absorbance at 670 nm [59].

#### 4.4.3. CCK-8 Reductive/Metabolic Activity Assay

Cellular reductive/metabolic activity was evaluated using a Cell Counting Kit-8 assay (CCK-8; Beyotime Biotechnology, Shanghai, China; Cat. No. C0037). VC and ED LMH cells were seeded into 96-well plates at equal densities.

At 6, 12, 24, 36, and 48 h after seeding, 10 μL of CCK-8 solution was added to each well containing 100 μL of culture medium, followed by incubation at 37 °C according to the manufacturer’s instructions. Absorbance was measured at 450 nm. OD_450_ values were used as an indicator of CCK-8-dependent cellular reductive/metabolic activity.

Where multiple technical wells were used for the same independently prepared cell sample, their OD_450_ values were averaged to obtain one value for the corresponding biological replicate. Individual wells derived from the same cell preparation were not treated as independent biological replicates.

#### 4.4.4. DCFH-DA Oxidation-Sensitive Fluorescence Assay

Intracellular oxidative status was assessed using the oxidation-sensitive DCFH-DA probe (Solarbio, Beijing, China; Cat. No. CA1410). VC and ED cells were seeded at equal densities and incubated with DCFH-DA working solution at 37 °C for 30 min in the dark. Cells were washed three times with serum-free culture medium and immediately imaged using a confocal microscope with excitation at 488 nm and emission at 525 nm. All image-acquisition parameters were kept identical between the VC and ED groups.

For each biological replicate, at least five randomly selected microscopic fields were analyzed. Fluorescence intensity was measured using ImageJ (v2.0.0) after background subtraction, using the same image-processing parameters for all samples. The measurements obtained from multiple fields were averaged to generate one value for the corresponding biological replicate. Individual microscopic fields were not treated as independent biological replicates.

### 4.5. Transcriptomic and Functional Enrichment Analyses

RNA sequencing was performed using experimental series A, comprising three independently transfected and GFP-sorted biological replicates per group.

VC and ED cells were harvested in parallel at the same predefined elapsed time after seeding. At the time of RNA collection, cultures in both groups had reached greater than 70% confluence. Total RNA was extracted using TRNzol reagent (TIANGEN Biotech, Beijing, China; Cat. No. DP424). RNA concentration and purity were assessed using a NanoDrop spectrophotometer (Thermo Fisher Scientific, Waltham, MA, USA), and RNA integrity was evaluated using an Agilent 2100 Bioanalyzer (Agilent, Singapore). Only RNA samples with an RNA integrity number (RIN) ≥ 8.0 were used for library construction.

Poly(A)+ mRNA was enriched using oligo(dT) magnetic beads and fragmented to approximately 200 bp. The fragmented mRNA was subjected to first- and second-strand cDNA synthesis, end repair, A-tailing, adapter ligation, and PCR amplification. The resulting libraries were sequenced on an Illumina NovaSeq 6000 (Illumina, Singapore) platform to generate paired-end 150 bp reads.

Raw reads were filtered using Trimmomatic (v0.39) to remove adapter sequences and low-quality reads [60]. Clean reads were aligned to the *Gallus gallus* GRCg7b reference genome using HISAT2 (v2.2.1) [61], and gene-level read counts were generated using featureCounts (v2.0.5) [62]. Library size, clean-read percentage, Q30 percentage, and genome-mapping rate were summarized for each sample. FPKM values were calculated for expression visualization. Principal component analysis was performed using log_2_(FPKM + 1)-transformed expression values of the 500 genes with the highest variance across all samples to assess the overall relationships among biological replicates and the separation between the VC and ED groups. Differential expression analysis between ED and VC cells was performed using raw gene-level counts with edgeR (v4.6.2) in R (v4.2.0) [63]. Genes with an absolute log2(Fold change) ≥ 1 and a Benjamini–Hochberg false discovery rate (FDR) < 0.05 were defined as differentially expressed genes (DEGs). Genes with log2(Fold change) > 0 were considered upregulated in ED cells, whereas genes with log2(Fold change) < 0 were considered downregulated.

Functional enrichment analysis was performed using g:Profiler (accessed on 18 June 2026), with *Gallus gallus* specified as the target organism [64]. Upregulated and downregulated DEGs were analyzed separately. Gene Ontology biological process, molecular function, and cellular component terms, together with KEGG pathway terms, were considered significantly enriched at FDR < 0.05. Representative enriched terms were visualized according to adjusted statistical significance, gene ratio, and the number of mapped query genes.

#### 4.5.1. Enrichment-Term Association Network

An enrichment-term association network was constructed using significantly enriched GO and KEGG terms identified from the downregulated differentially expressed gene set. The degree of gene overlap between each pair of enriched terms was quantified using the Jaccard index:

$$J(A,B)\;=\;\frac{\left|A\cap B\right|}{\left|A\cup B\right|}$$

where *A* and *B* represent the query-gene sets associated with two enriched terms. Term pairs with a Jaccard index ≥0.10 were retained as gene-overlap associations.

Direct parent–child relationships among GO biological process, cellular component, and molecular function terms were obtained from the GOBPPARENTS, GOCCPARENTS, and GOMFPARENTS mappings in the GO.db package, respectively, and were incorporated into the network as directed GO hierarchy edges.

For each enriched term, network association strength was defined as the sum of the weights of all retained Jaccard association edges connected to that term. GO hierarchy edges were not included in the calculation of association strength. To improve network readability, only enriched terms with an association strength >0.5 and more than five mapped query genes were retained for visualization. The ten terms with the highest association-strength values were designated as hub terms and highlighted in the network.

The network was constructed and visualized using the igraph and ggraph packages in R. Node shape represented the enrichment-term category, node size represented association strength, node fill represented the number of mapped query genes, and edge width represented the Jaccard index. Initial node coordinates were generated using the Fruchterman–Reingold force-directed layout. Selected node positions were subsequently adjusted using vis Network to improve label readability. This coordinate adjustment affected only the graphical layout and did not alter network nodes, edges, edge weights, or topology.

#### 4.5.2. Gene Set Enrichment Analysis

Gene set enrichment analysis (GSEA) was conducted using the ranked gene list derived from the ED-versus-VC comparison [65]. Two complementary approaches were applied.

First, GSEA was performed using the curated gene set collection C2 for *Gallus gallus* obtained through the msigdbr package (v7.5.1). Second, the gseKEGG function implemented in clusterProfiler (v4.6.0) was applied using *Gallus gallus* KEGG gene sets [66]. Gene sets with an FDR < 0.05 were considered significantly enriched.

#### 4.5.3. STRING Functional Association and MCODE Analyses

*HMOX1* and the core enrichment genes contributing to the selected negatively enriched KEGG pathways were submitted to STRING v12.0 using *Gallus gallus* as the background organism. The full STRING functional-association network was retrieved using a minimum required combined interaction score of 0.7, corresponding to high confidence.

All available evidence channels, including text mining, experiments, curated databases, co-expression, gene neighborhood, gene fusion, and phylogenetic co-occurrence, were enabled.

The resulting functional-association network was imported into Cytoscape (v3.9.1) for visualization. Densely connected subnetworks were identified using the MCODE plugin (v2.0.3) with the following parameters: degree cutoff = 2, node score cutoff = 0.2, K-core = 2, and maximum depth from seed = 100. Self-loops were excluded, Haircut was enabled, and Fluff was disabled. The two highest-scoring clusters containing at least three nodes were selected for visualization.

### 4.6. RT-qPCR Validation Using an Independent Biological Sample Set

Selected RNA-seq expression patterns were validated by RT-qPCR using an independent biological sample set comprising three VC and three ED biological replicates. Each replicate was derived from an independently transfected, GFP-sorted, and recovered LMH cell preparation. VC and ED cells were harvested in parallel at the same elapsed time after seeding, when cultures in both groups had reached greater than 70% confluence.

Total RNA was extracted using TRNzol reagent (TIANGEN Biotech, Beijing, China; Cat. No. DP424) and reverse-transcribed using a TIANGEN cDNA synthesis kit (TIANGEN Biotech, Beijing, China; Cat. No. KR116). RT-qPCR was performed using a TIANGEN SYBR Green-based quantitative PCR kit according to the manufacturer’s instructions (TIANGEN Biotech, Beijing, China; Cat. No. FP313). The genes examined included *IRF1*, *JUN*, *NFKBIA*, *PTGS2*, *PCNA*, *MCM2* and *IL1B*. *ACTB* was used as the endogenous reference gene, and primer sequences are provided in Table S6 [67,68,69,70,71,72,73]. Relative expression was calculated using the 2^−ΔΔCt^ method, with the VC group used as the calibrator.

### 4.7. Statistical Analysis

The initial sgRNA screening was performed using a single transfection batch and did not include biological replicates. The resulting TA-cloning data were used only to estimate and compare clone-level editing proportions among the three sgRNAs.

All experiments used for phenotypic characterization were performed using biological replicates generated through independent transfection and GFP-positive cell-sorting procedures. Experimental series A provided three biological replicates per group for HO-1 protein quantification, RNA sequencing, and RT-qPCR analysis. Experimental series B provided four biological replicates per group for the heme, biliverdin, CCK-8, DCFH-DA oxidation-sensitive fluorescence assays and RT-qPCR.

Data are presented as the mean ± standard deviation (SD), as indicated in the corresponding figure legends. Comparisons between two groups were performed using unpaired two-tailed Student’s *t*-tests. For the CCK-8 assay, differences between the VC and ED groups were evaluated separately at each measurement time point using unpaired two-tailed Student’s *t*-tests. This analysis was used to determine whether the CCK-8 signal differed between the two groups at each individual time point.

For transcriptomic analysis, *p*-values were adjusted for multiple comparisons using the Benjamini–Hochberg FDR procedure, and FDR < 0.05 was used as the significance threshold. Statistical analyses of experimental data were performed using R (v4.2.0) or GraphPad Prism (v9.0). Statistical significance was defined as follows: * *p* < 0.05, ** *p* < 0.01, *** *p* < 0.001, and **** *p* < 0.0001.

## 5. Conclusions

This study established a heterogeneous CRISPR/Cas9-mediated *HMOX1*-edited cell population in chicken LMH cells and showed that reduced HO-1 protein abundance was accompanied by disruption of heme–biliverdin metabolic homeostasis. *HMOX1*-edited cells exhibited intracellular heme accumulation, reduced biliverdin levels, increased oxidation-sensitive fluorescence, and lower CCK-8 reductive/metabolic activity, indicating that reduced HO-1 abundance was associated with alterations in the avian heme–biliverdin axis and cellular redox state. Transcriptomic analysis further revealed coordinated remodeling of the basal transcriptional profile, characterized by repression of immune-, stress-, cytokine-, and extracellular signaling-associated programs together with increased expression of selected DNA replication- and cell-cycle-related genes. Enrichment-term association and STRING functional-association analyses identified a coordinated module involving *IL1B*, *JUN*, *NFKBIA*, *IRF1*, *TGFB1*, *IL10*, *CCL5*, and *PTGS2*. Together, these findings suggest that HO-1 contributes not only to heme degradation and biliverdin production but also to the maintenance of redox-associated and basal transcriptional homeostasis in chicken hepatic cells.

## Data Availability

The datasets generated and analyzed in this study have been deposited under BioProject accession PRJCA070174. Other data supporting the findings of this study are included in the article and its Supplementary Materials.

## Supplementary Materials

The following supporting information can be downloaded at https://www.mdpi.com/article/10.3390/ijms27167120/s1.

## Abbreviations

The following abbreviations are used in this manuscript:

ANOVA	Analysis of variance
CCK-8	Cell Counting Kit-8
DEG	Differentially expressed gene
ED	*HMOX1*-edited
ELISA	Enzyme-linked immunosorbent assay
FDR	False discovery rate
GO	Gene Ontology
GSEA	Gene set enrichment analysis
HO-1	Heme oxygenase-1
KEGG	Kyoto Encyclopedia of Genes and Genomes
LMH	Chicken hepatocellular carcinoma-derived cell line
PCA	Principal component analysis
PPI	Protein–protein interaction
ROS	Reactive oxygen species
sgRNA	Single guide RNA
VC	Vector-control
T7E1	T7 endonuclease I
WT	Wild type
