# Supplementary material for: Heterogeneous CRISPR/Cas9 Editing of HMOX1 Is Associated with Altered Heme–Biliverdin Metabolism and Basal Stress-Associated Transcriptional Programs in Chicken LMH Cells
Source: Int J Mol Sci. 2026 Aug 8;27(16):7120. doi: 10.3390/ijms27167120 (PMC13513460; doi:10.3390/ijms27167120)
Supplement: Supplementary file 1 [file ijms-27-07120-s001.zip › ijms-4431284-supplementary.pdf]

International Journal of
Molecular Sciences

Supplementary Materials

Heterogeneous CRISPR/Cas9 Editing of HMOX1 Is Associated with Altered Heme–Biliverdin Metabolism and Basal Stress-Associated Transcriptional Programs in Chicken LMH Cells

Haonan Tang 1,#, Huaiyu Li 1,#, Yurong Tai 1,2, Xue Yang 1, Letian Zhang 1, Yuhao Ma 1, Ganxian Cai 1, Hongyang Zhao 1, Tong Zeng 1, Xiaohua Ai 1, Shuang He 1, Jiankui Wang 1, Zhiliang Gu 3 and Xuemei Deng 1,2,*

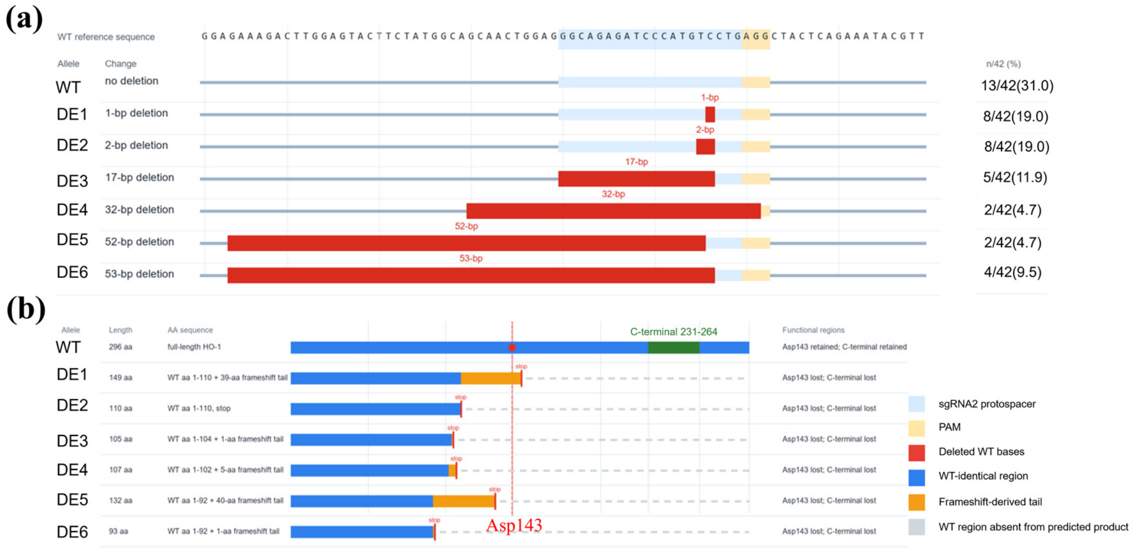

Figure S1. Sequence composition and predicted coding consequences of the *HMOX1* deletion alleles identified in the initial sgRNA2 screening population. (a) Alignment of the WT *HMOX1* target sequence and the six distinct deletion alleles, designated DE1 – DE6, identified by TA cloning and Sanger sequencing. The 29 edited clones comprised these six deletion alleles, containing deletions of 1, 2, 17, 32, 52, and 53 bp, respectively. The sgRNA2 protospacer and protospacer-adjacent motif (PAM) are highlighted in light blue and yellow, respectively, and deleted WT nucleotides are indicated in red. The number and proportion of clones carrying each allele are shown on the right. (b) Predicted coding consequences of DE1 – DE6 following reconstruction and in silico translation of the corresponding mutant HMOX1 coding sequences. The WT HO-1 protein comprised 296 amino acids, whereas the inferred translation products of DE1 – DE6 comprised 149, 110, 105, 107, 132, and 93 amino acids, respectively. Blue segments indicate amino acid sequences identical to WT HO-1, orange segments indicate non-native frameshift-derived sequences, red marks indicate predicted premature termination sites, and gray dashed segments indicate WT regions absent from the inferred translation products. The position of the native catalytic residue Asp143 and the C-terminal region spanning amino acids 231 – 264 are indicated. All six deletion alleles were predicted to lack the native Asp143 residue and the C-terminal region.

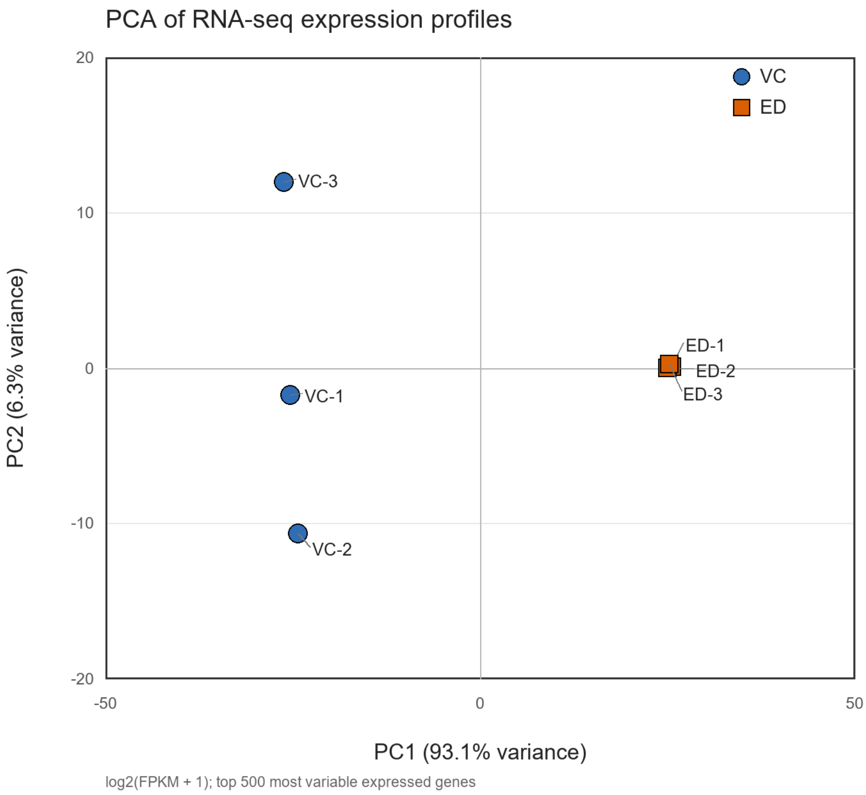

Figure S2. Principal component analysis of RNA-seq expression profiles in vector-control and *HMOX1*-edited LMH cells. Principal component analysis (PCA) was performed using $\log_2$(FPKM + 1)-transformed expression values of the 500 most variable expressed genes. Each point represents one independently transfected and GFP-sorted biological replicate (n = 3 per group). Blue circles indicate vector-control cells (VC), and orange squares indicate HMOX1-edited cells (ED). PC1 and PC2 accounted for 93.1% and 3.3% of the total variance, respectively, with VC and ED samples separating primarily along PC1.

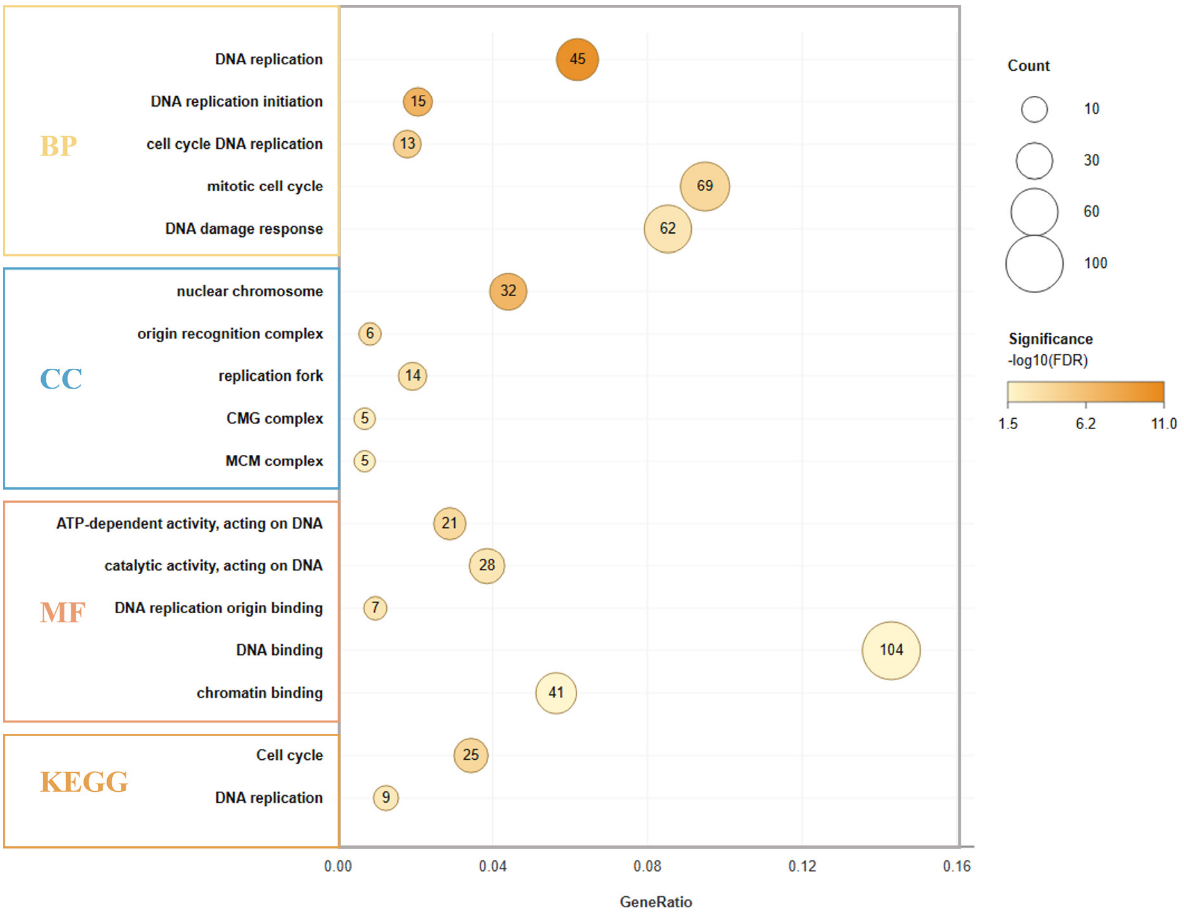

Figure S3. Functional enrichment of upregulated genes in *HMOX1*-edited LMH cells. GO biological process (BP), cellular component (CC), molecular function (MF), and KEGG terms are shown. Bubble size indicates gene count, color indicates -log10(FDR), and the x-axis indicates gene ratio.

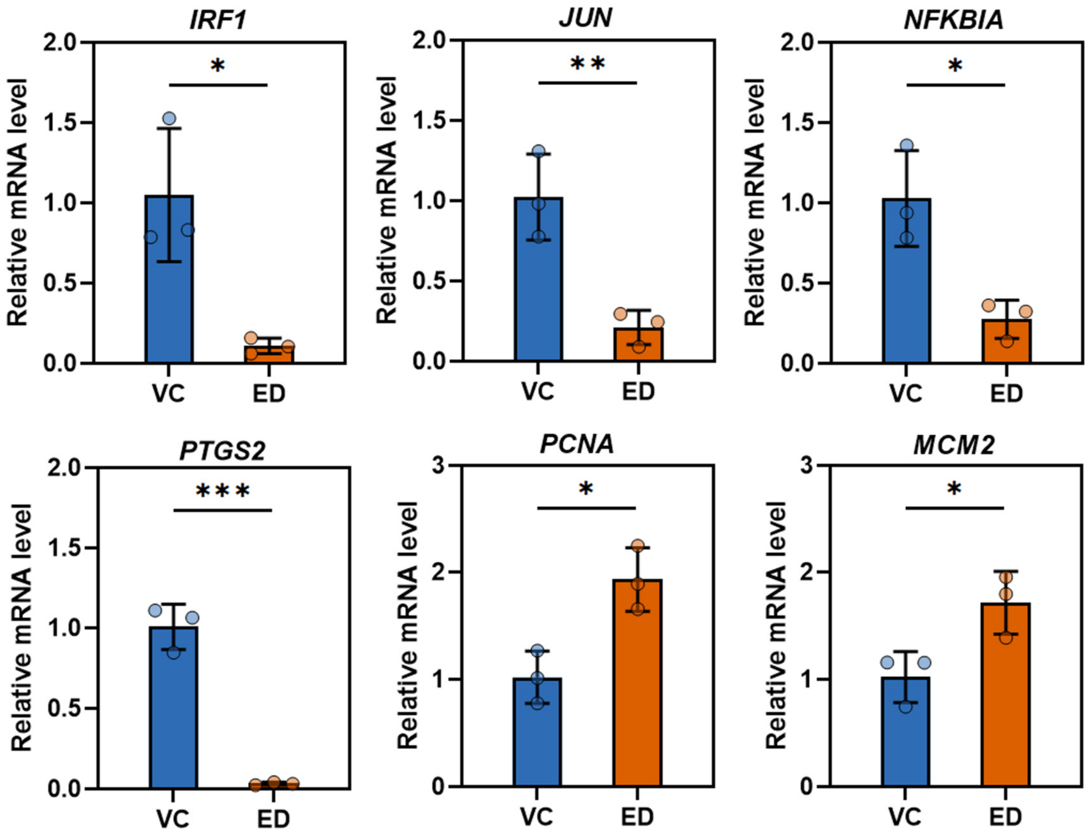

Figure S4. RT-qPCR validation of selected RNA-seq expression patterns in an independent biological sample set. Relative expression levels of *IRF1*, *JUN*, *NFKBI*A, *PTGS2*, *PCNA*, and *MCM2* in vector-control (VC) and *HMOX1*-edited (ED) LMH cells were determined using RT-qPCR. Expression was normalized to *ACTB* and calculated using the $2^{-\Delta\Delta Ct}$ method. Data are presented as the mean ± SD of three biological replicates per group, with individual points representing biological replicates. Statistical significance was assessed using unpaired two-tailed Student's t-tests based on ΔCt values. *P < 0.05; **P < 0.01; ***P < 0.001.

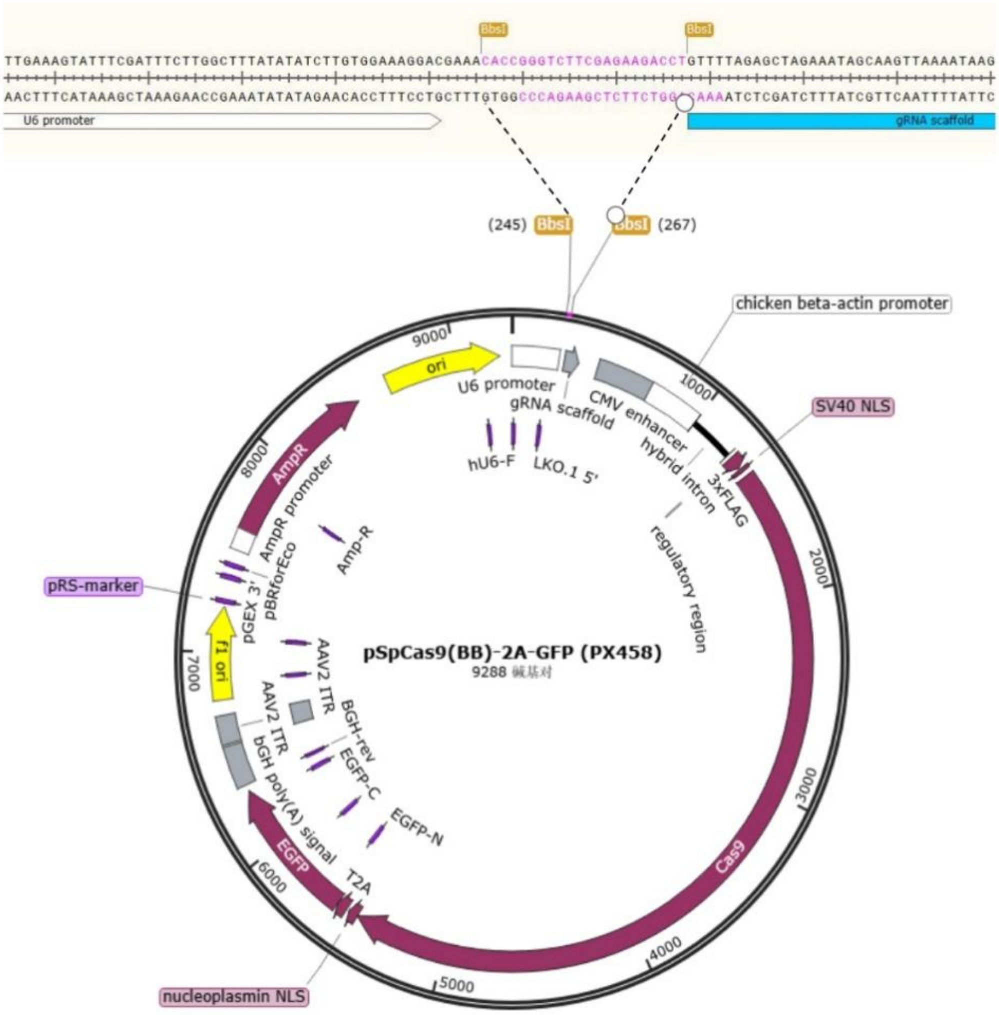

Figure S5. Plasmid map of PX458.

Table S1. AlphaFold3 model confidence metrics for WT HO-1 and representative DE1-DE6 predicted translated products

Model	Length (aa)	ranking score	pTM	mean pLDDT	fraction disordered	clash
WT	296	0.9	0.76	76.92	0.27	no
DE1	149	0.81	0.69	72.56	0.24	no
DE2	110	0.92	0.81	89.93	0.23	no
DE3	105	0.91	0.8	89.98	0.23	no
DE4	107	0.89	0.77	86.68	0.23	no
DE5	132	0.78	0.61	67.86	0.34	no
DE6	93	0.9	0.76	88.23	0.28	no

Note: WT HO-1 and the inferred amino-acid sequences encoded by six representative Sanger-confirmed *HMOX1* deletion alleles were submitted separately to AlphaFold3 as single-chain protein inputs. For each sequence, the top-ranked model was selected according to the AlphaFold3 ranking score. pTM indicates confidence in the predicted global fold, whereas mean pLDDT reflects average residue-level local confidence. Fraction disordered indicates the proportion of residues predicted to be disordered or flexible. Clash

indicates whether obvious structural clashes were reported in the selected model. These models were used only to visualize predicted structural consequences of representative edited alleles and do not demonstrate stable expression of truncated proteins in the sgRNA2-edited cell pool.

Table S2. sgRNA sequences and cloning primers.

Name	sgRNA sequences	Forward Primer	Reverse Primer
sgRNA 1	GTACTCCAAGTCTTTCT CCA	CACCGG-TACTCCAAGTCTTTCTCCA	AAACTGGAGAAAGACTT-GGAGTAC
sgRNA 2	GGCAGA-GATCCCATGTCCTG	CACCGGGCAGA-GATCCCATGTCCTG	AAACCAGGACATGG-GATCTCTGCC
sgRNA 3	GATGCTTCTTGCCAAC-GACG	CACCGGATGCTTCTTGCCAAC-GACG	AAACCGTCGTTGGCAAGAA-GCATC

Table S3. Preparation and allocation of cell samples used in this study

Series	Group	n	Sample IDs	Preparation IDs (T/S)	Post-sort culture	Assays
Screening	sgRNA1	1	sgRNA1	T01-sg1 / S01-sg1	24 h; P0	TA cloning, Sanger sequencing
Screening	sgRNA2	1	sgRNA2	T01-sg2 / S01-sg2	24 h; P0	TA cloning, Sanger sequencing
Screening	sgRNA3	1	sgRNA3	T01-sg3 / S01-sg3	24 h; P0	TA cloning, Sanger sequencing
Series A	ED	3	ED-A1–A3	T02-ED-1–3 / S02-ED-1–3	48 h; P1	RNA-seq, HO-1 ELISA
Series A	VC	3	VC-A1–A3	T02-VC-1–3 / S02-VC-1–3	48 h; P1	RNA-seq, HO-1 ELISA
Series B	ED	4	ED-B1–B4	T03-ED-1–4 / S03-ED-1–4	72 h; P2	Heme, biliverdin, CCK-8, DCFH-DA, RT-qPCR
Series B	VC	4	VC-B1–B4	T03-VC-1–4 / S03-VC-1–4	72 h; P2	Heme, biliverdin, CCK-8, DCFH-DA, RT-qPCR

Note: T01, T02, and T03 indicate experimental rounds rather than individual transfection procedures. Within Series A and Series B, each sample was generated from an independently cultured LMH cell preparation and underwent a separate transfection, GFP-positive cell sorting, and post-sorting recovery procedure. No cells were pooled between biological replicates. In Series A, each sample was divided into two paired aliquots at harvest: one for RNA-seq and the other for HO-1 protein quantification. In Series B, heme and biliverdin were measured using first-passage cultures, whereas CCK-8 and DCFH-

DA assays were performed using second-passage cultures derived from the corresponding independent cell preparations.

Table S4. Prediction of off-target sites

Name	Location	Bulge Size	Mismatches	Sequence
TS	chr1-52145778	0	0	GGCAGAGATCCCATGTCCT
OT-1	chr1-51952376	1	0	GGgCAGAGATCCCATGTCCTG
OT-2	chrZ-17471387	1	2	GGCAGCAatTCCCATGTCCTG
OT-3	chr8-19033177	1	2	GGCAGAGGATCCCcTGTCCcG
OT-4	chr1-114062894	1	2	GGaAGAGATCCTCATtTCCTG
OT-5	chr11-17988295	1	2	GGCAGAGAgCCCATGgCGCTG
OT-6	chr2-120446254	2	2	GaCAaAGGAATCCCATGTCCTG
OT-7	chr8-13887	2	2	GGCAGAGATCCCATGAAagCTG

Note: TS represents the target site, OT represents the off-target site, Location represents the chromosome where the off-target site is located, Bulge Size represents the number of bases protruded, Mismatches represents the number of mismatched bases, and Sequence represents the base sequence, in which yellow is the bases protruded compared with the target sequence. The red marks are mismatched bases. Abbreviations: ED, sgRNA2-edited cell population; VC, empty-vector control; T, transfection; S, fluorescence-activated cell sorting; P0–P2, number of passages after sorting.

Table S5. Validation and off-target primer sequences.

Name	Forward Primer	Reverse Primer
YW1	TAGGCTCCCAGTCTGGACAA	ACTGGGTGTGCATGAG-TAACTT
YW2	GGCTGGCTGGTTGTTACTCA	TGCTACACAAGAAACCTG-CAAC
OT-1	GTCGTCCAGGTAAGCGGA	AAAACCCGCGGTACAAGGTA
OT-2	AGTCTGGCCACGTGCTATCAT	GCAAAAGGTGAAGCTAGGCG
OT-3	CGGTACTGCGGACATGACTT	ACCCTGACATGCATACCCAC
OT-4	TTTGTGGGCTCTCTGTGTCA	TACCCACTCACATTTCACGCA

OT-5	CATTTTCAGGTCGCTCGCAC	GTTTGAGGTCTGAGCGTTGC
OT-6	CGGAAAGACCCAAAAGGGGA	ACGTACCCACTGGACTGAGA
OT-7	AGCCAGCCCAAACAAACTCA	TTACCTTTTGGGTGGGAGTGG

Table S6. Primers used for RT-qPCR.

Gene	Forward Primer (5'-3')	Reverse Primer (5'-3')
JUN [67]	CTCATCATCCAGTCCAGCAA	TGTTCTGGTTGTGCAGTTCC
IL1B [68]	TGCCTGCAGAAGAAGCCTCG	CTCCGCAGCAGTTTGGTCAT
IRF1 [69]	GCTACACCGCTCACGA	TCAGCCATGGCGATTT
NFKBIA [70]	CACCAACTACAACGGCCATA	TGAAGGTCTACGGCCAAGTG
PTGS2 [71]	CTGCTCCCTCCCATGTCAGA	CACGTGAAGAATTCCGGTGTT
MCM2 [72]	TTGCGATCACTCAGGCAACT	TCCTGGTTCTGGGACTGGAA
PCNA [73]	GCAGATGTTCCTCTCGTTGTGGAG	GAGCCTTCCTGCTGGTCTTCAATC
ACTB [72]	GCCCCCAAAGTTCTACAAT	AGGCGAGTAACTTCCTGTA

Supplementary Datasets

Dataset S1. Differentially expressed genes and FPKM values (Additional File 1.csv).

Dataset S2. GO enrichment results (Additional File 2.xls).

Dataset S3. KEGG pathway enrichment results (Additional File 3.xls).

Dataset S4. GSEA results (Additional File 4.csv).
